# Bridging the data gaps in the epidemiology of hepatitis C virus infection in Malaysia using multi-parameter evidence synthesis

**DOI:** 10.1186/s12879-014-0564-6

**Published:** 2014-11-07

**Authors:** Scott A McDonald, Rosmawati Mohamed, Maznah Dahlui, Herlianna Naning, Adeeba Kamarulzaman

**Affiliations:** Centre of Excellence for Research in AIDS (CERiA), University of Malaya, Kuala Lumpur, Malaysia; Centre for Infectious Disease Control, National Institute for Public Health and the Environment, Bilthoven, 3720BA The Netherlands; School of Health & Life Sciences, Glasgow Caledonian University, Cowcaddens Road, Glasgow, Scotland; Faculty of Medicine, University of Malaya, Kuala Lumpur, Malaysia; Julius Centre, Faculty of Medicine, University of Malaya, Kuala Lumpur, Malaysia

**Keywords:** Hepatitis C virus, Prevalence, Bayesian evidence synthesis, People who inject drugs, Malaysia

## Abstract

**Background:**

Collecting adequate information on key epidemiological indicators is a prerequisite to informing a public health response to reduce the impact of hepatitis C virus (HCV) infection in Malaysia. Our goal was to overcome the acute data shortage typical of low/middle income countries using statistical modelling to estimate the national HCV prevalence and the distribution over transmission pathways as of the end of 2009.

**Methods:**

Multi-parameter evidence synthesis methods were applied to combine all available relevant data sources - both direct and indirect - that inform the epidemiological parameters of interest.

**Results:**

An estimated 454,000 (95% credible interval [CrI]: 392,000 to 535,000) HCV antibody-positive individuals were living in Malaysia in 2009; this represents 2.5% (95% CrI: 2.2-3.0%) of the population aged 15-64 years. Among males of Malay ethnicity, for 77% (95% CrI: 69-85%) the route of probable transmission was active or a previous history of injecting drugs. The corresponding proportions were smaller for male Chinese and Indian/other ethnic groups (40% and 71%, respectively). The estimated prevalence in females of all ethnicities was 1% (95% CrI: 0.6 to 1.4%); 92% (95% CrI: 88 to 95%) of infections were attributable to non-drug injecting routes of transmission.

**Conclusions:**

The prevalent number of persons living with HCV infection in Malaysia is estimated to be very high. Low/middle income countries often lack a comprehensive evidence base; however, evidence synthesis methods can assist in filling the data gaps required for the development of effective policy to address the future public health and economic burden due to HCV.

**Electronic supplementary material:**

The online version of this article (doi:10.1186/s12879-014-0564-6) contains supplementary material, which is available to authorized users.

## Background

The burden of hepatitis C virus (HCV) infection and its epidemiology in Malaysia are still to be documented. Although several studies of drug-using populations and clinical series of HCV patients in Malaysia have been conducted [1]-[5], very little is known regarding the population-level prevalence and the distribution over transmission pathways [6].

The HIV/AIDS epidemic in Malaysia has drawn considerable governmental and public support, with comprehensive screening, harm reduction services for people who inject drugs (PWID, who represent 67% of reported cases as of 2012 [7]), and antiretroviral therapy (ART) treatment now widely available. HIV is now recognised as being under control [7]. Harm reduction initiatives aimed at reducing HIV transmission were rolled out in 2005 and have been rapidly expanded since. Although these initiatives have had added benefit for HCV by virtue of addressing shared routes of transmission, resources for HCV testing and treatment are lacking in comparison with those for HIV.

HCV antibody testing is currently carried out in blood donor sites, opiate substitution treatment (OST) and drug rehabilitation centres, prison, antenatal clinics, hospital and general practice settings; however, screening can only be considered comprehensive for prospective blood donors. A system for notification of HCV-infected individuals to the Ministry of Health has been in place since 2003, but notifications are mostly from opportunistic screening as described above, and thus cannot serve as an indicator of incidence. As of the end of 2013, a cumulative total of 12,380 HCV cases have been notified (Disease Control Division, Ministry of Health, pers. comm.).

Malaysia has a recognised illicit drug use problem, with injecting drug use alarmingly prevalent historically. The National Anti-Drugs Agency (NADA) reported approximately 328,500 drug users registered (i.e., tested positive and arrested) in the period 1988-2009. Drug preferences and practices fluctuate over time, but 32-42% of all registrations in the period 2005-2009 were associated with heroin [8]. Although injecting drug use is considered by infectious disease experts to be the dominant mode of HCV transmission in Malaysia, consistent with data on risk distributions in a number of developed countries [9], no evidence exists to support this presumption. Available data on the distribution over transmission routes is primarily from clinical series and other selected populations [3],[5],[6]; the selection bias inherent in this type of study is well known [9].

Although the eventual aim of eradicating HCV globally may be possible, there are many public health challenges that first have to be met at the national level [10]. An integral strategy of the WHO’s Framework for Global Action on Viral Hepatitis [11] is the development of evidence-based policy, and the WHO has recently published guidelines for screening and treatment of HCV, [12] with special attention on prioritising the resources available to low- and middle-income countries. Given the lack of a comprehensive HCV evidence base in Malaysia, methods for overcoming the existing data gaps would be of unquestionable value for planning the public health response to the HCV epidemic.

The goal of this paper is to estimate the HCV antibody-positive (Ab+) prevalence, and the distribution over transmission routes, of the infected population in Malaysia using statistical modelling to combine of all of the relevant evidence. A better understanding of the epidemiology of HCV is crucial to the development and deployment of effective prevention and screening programmes, and for forecasting trends in HCV-related disease.

## Methods

Estimation of key epidemiological indicators (i.e., HCV Ab+ prevalence, number of HCV Ab+ infected persons, proportions with PWID and non-PWID risk among HCV Ab+ persons) was conducted within the multi-parameter evidence-synthesis (MPES) framework, a recently developed modelling approach useful for combining multiple data sources to derive estimates for indicators that cannot easily be measured directly [13]-[15]. In this approach, a model is specified for the relationships between the various data sources and the parameters of interest, and estimation is typically carried out using Markov-Chain Monte-Carlo (MCMC) sampling methods. Prior distributions for parameters can be specified as informative or vague, and the task of the model is to compute the posterior distributions for all parameters. Importantly, uncertainty is correctly propagated through the model, and can be expressed as 95% credible intervals (CrIs).

We related the main parameters of interest using Bayes’ Theorem (Eq. 1 below). In this equation, the sole parameter for which no direct data were available - the probability of PWID risk given HCV Ab+ status, or *P*(PWID|HCV+) - can be expressed if the other probabilities in the equation are known, or can themselves be estimated from available data [16],[17].

1 $$P\left(\mathrm{PWID}|HCV^{+}\right)=\frac{P\left(HCV^{+}|\mathrm{PWID}\right)P\left(\mathrm{PWID}\right)}{P\left(HCV^{+}\right)}$$

**Figure 1:**
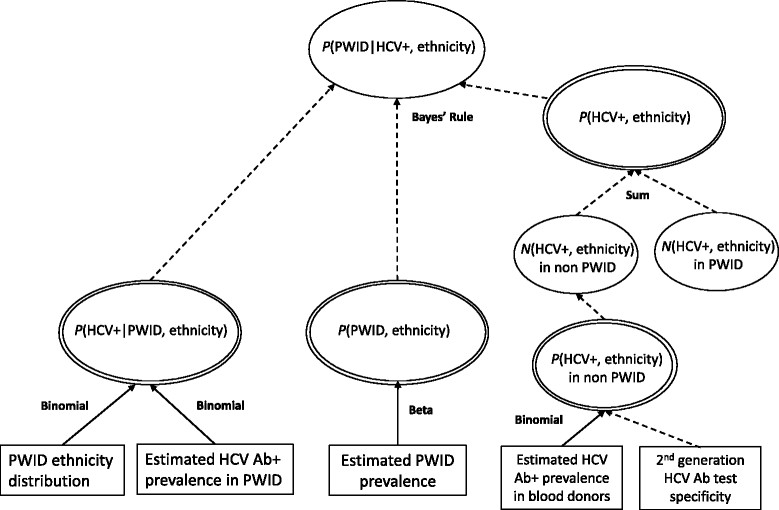
**The multi-parameter evidence synthesis model (depicted as a directed acyclic graph [DAG]) used to estimate the prevalence of HCV infection and the proportion of HCV Ab+ individuals with PWID and non-PWID modes of transmission.** Stratification is by ethnicity and sex, but for clarity the DAG for males only is shown. Ovals indicate model parameters; rectangles indicate data sources. Distributional and functional relationships between parameters/data sources are indicated by solid and dashed arrows, respectively.

Figure 1 shows the MPES model structure, and lists the data sources informing each of the three probabilities required - *P*(HCV+ |PWID), *P*(HCV+), and *P*(PWID) - to estimate *P*(PWID|HCV+) and the relevant stratified subpopulation sizes. See Additional file 1: Appendix B for MPES model equations and prior distributions. All inputs and estimated values are with respect to the adult population aged 15-64 years only.

### Data sources and model parameters

As a preliminary step, we conducted a thorough review of both the published and grey literature on HCV in Malaysia, and compiled a list of data availability and data gaps regarding the prevalence and incidence of HCV, HCV-HIV co-infection, and the demographic characteristics of the subpopulations at risk of acquiring infection. See Table 1 for more detailed information on the data sources contributing to the evidence synthesis, including the actual counts or proportions used as inputs to the MPES model. The contributing data sources represent a wide range of years (1991-2011); however, as most sources collected data within the period 2006-2011, the evidence synthesis is assumed to apply to the year 2009.

**Table 1:** **Data sources and values for informing the MPES model parameters**

Model parameter	Data	Source
*P*(HCV|PWID, Malay)	293/427 (68.6%)	PWID not in treatment; calculated from Reference [2]
*P*(HCV|PWID, Chinese)	27/45 (60.6%)	
*P*(HCV|PWID, Other)	33/54 (60.6%)	
*P*(PWID)	2.22%	Multi-state Markov model (Additional file 1: Appendix A)
*Males*	97.6% of *P*(PWID)	Criminal justice data [26]
*P*(PWID, Malay)	427/526 (81.2%)	Ethnicity distribution of PWID from Reference [2]
*P*(PWID, Chinese)	45/526 (8.6%)	
*P*(PWID, Other)	54/526 (10.3%)	
*Females*	2.4% of *P*(PWID)	Criminal justice data [26]
*Males*		
*P*(HCV|nonPWID, Malay)	28/1713 (1.63%)	Blood donors [19]
*P*(HCV|nonPWID, Chinese)	22/1373 (1.60%)	
*P*(HCV|nonPWID, Other)	3/454 (0.66%)	
*Females*		
Ratio (φ) of: *P*(HCV|nonPWID, female) to *P*(HCV|nonPWID, male)	55/1716 [males]	Blood donors in Thailand [23]
	8/451 [females]	
	200/51414 [males]	Blood donors in Singapore [22]
	41/13758 [females]	
*P*(HIV|HCV)	40/113 (35%)	PWID in the Malaysian fisherman community [28]

We specified a model in which most parameters were stratified by sex as well as ethnicity (i.e., Malay, Chinese, Indian/other), as the baseline risks of being an [injecting] drug user, and of being HCV Ab+ given PWID status appear to vary according to ethnicity [2],[8]. Variation in HCV prevalence may be due to ethnic differences in drug-taking and injecting equipment sharing behaviours [1]; for instance, ethnic Chinese in Singapore were reported to share drugs much less often than other ethnicities [18].

#### P(HCV+): probability of HCV

The overall probability of HCV Ab+ in the population is defined as the weighted sum of two probabilities: the probability of being HCV Ab+ given PWID risk weighted by the probability of having PWID risk, and the probability among individuals with non-PWID risk. The population probability of non-PWID risk is defined as (1 - *P*(PWID)):

$$P\left(HCV^{+}\right)=P\left(HCV^{+}|\mathrm{PWID}\right)P\left(\mathrm{PWID}\right)+P\left(HCV^{+}|\mathrm{nonPWID}\right)P\left(\mathrm{nonPWID}\right)$$

#### P(HCV+ |nonPWID): probability of HCV in persons with non-PWID risk

To estimate the probability of HCV infection in individuals with non-PWID risk, two blood donor studies were available [19],[20]. The first is a large study of donor blood specimens collected in 1991-1992 in Kuala Lumpur, which found an overall HCV seroprevalence of 1.49% [19]; these data were considered relevant evidence for the synthesis. A more recent study (2008-2009) conducted in north-eastern Malaysia reported a HCV seroprevalence of 0.45% [20]. These data were not used, as donor pre-screening at this time was much more stringent compared with the early 1990s, and thus there is a risk of greatly under-estimating the prevalence in non-PWID.

The selected study reported seroprevalence separately by ethnic group. We adjusted the reported seroprevalence for the specificity (*Spec* =99.7%) of the second-generation assay employed [21] according to the functional relationship between observed prevalence and true prevalence (see Additional file 1: Appendix B).

In this study, the vast majority of the tested population was male - only 29 females among 3,540 persons tested - and there were no positive specimens among the females. Therefore, it may be incorrect to generalise male prevalence to females. As a solution, the female:male positivity ratios from studies of blood donors in neighbouring countries (Singapore [22] and Thailand [23]) were included to inform a model parameter that defines the female prevalence as a proportion of the prevalence among males. The Thai study population consisted of 2,167 blood donors (July 1999 to June 2000), with observed sex-specific HCV Ab prevalences of 3.21 and 1.77% for males and females, respectively [23]; the female:male positivity ratio was therefore 0.551. The Singapore study population consisted of 65,208 blood donors (December 1992 to August 1994), with observed sex-specific HCV Ab prevalences of 0.389 and 0.298% for males and females, respectively [22]; the female:male positivity ratio was therefore 0.766. These two ratios are effectively weighted by study size in the evidence synthesis model.

#### P(HCV+ |PWID): probability of HCV in PWID

Two studies were identified that reported the prevalence of HCV infection among PWID in Malaysia; one consisted of 159 participants enrolled on an agonist clinical trial [1] and the other was a large study (*n* = 526) of PWID in five urban areas who were not in treatment [2],[24]. The overall HCV prevalence in the two studies was 89.9% and 67.1%, respectively. Because the prevalence of HCV infection may be higher among participants recruited in a clinical or drug treatment setting, compared with the community setting, only the data from the latter study were incorporated in the evidence synthesis, as this study should be more representative of HCV prevalence among PWID in general. Prevalence could be determined according to ethnic group (Table 1). Because 95% of the study participants were male, there were insufficient data to infer the presence of sex differences, and so HCV prevalence in female PWID was assumed to be identical to prevalence in male PWID.

#### P(PWID): probability of PWID in the general population

*P*(PWID) was estimated using a separate model to simulate the population prevalence of PWID as the sum of the sizes of the cumulative total living active PWID and ex-PWID subpopulations in 2009. An estimate of the active PWID population size - 170,000 in 2009 - had been derived using expert consensus [25], but the size of the ex-PWID population is also required, as ex-PWID may also be HCV-infected. We developed a simple individual-based multi-state Markov model of the total PWID population, under some basic assumptions. Because the consensus estimate refers to the prevalence of living active (or current) PWID, the value of 170,000 was used for model fitting only. A detailed description of this model, the fitting procedure, and the results are provided in the Additional file 1: Appendix A. The probability of being PWID was estimated stratified by ethnicity and sex. The probability of being PWID according to ethnicity was informed by the distribution over ethnic groups among the PWID in a community-based study of PWID [2], and the probability among females was set to 2.4% of the total *P*(PWID), which is based on NADA figures [26].

#### P(PWID|HCV+): probability of PWID risk among HCV Ab+ persons

No data were available regarding the distribution over probable routes of transmission among HCV Ab+ individuals in the general population. However, the unknown probability of PWID risk given HCV Ab+ status, or *P*(PWID|HCV+), stratified by sex and ethnicity, can be inferred using Bayes’ Theorem (Eq. 1, above). Equivalently, it was computed as a proportion: the estimated number of HCV+ persons with PWID risk divided by the estimated total HCV+ persons. Similarly, the probability of non-PWID risk given HCV Ab+ status was calculated as the estimated number of HCV+ persons with non-PWID risk divided by the estimated total HCV+ persons.

#### P(HIV+ |HCV+): prevalence of HIV co-infection in HCV Ab+ persons

HIV and HCV share transmission routes, and HIV co-infection is common in Malaysian PWID and other drug users; one study reports a HIV co-infection rate of 43% among HCV-infected drug users not in treatment [2]. HIV co-infection can lead to a lower rate of spontaneous viral clearance, accelerated disease progression, and worse outcomes following antiviral therapy [27]. The prevalence of HIV co-infection among HCV Ab+ individuals does not influence the other parameters in the MPES model and can easily be estimated. A recent study in the Malaysian fishermen community found that 35% of HCV Ab+ were HIV co-infected [28]; the data underlying this prevalence value were incorporated in the MPES model.

#### Prevalence of chronic HCV infection

We estimated the size of the living chronically infected population by incorporating an estimate from a recent systematic review [29] indicating that 26% of acute infections spontaneously resolve into the MPES model. As with the computation of *P*(HIV+ |HCV+), including this parameter had no influence on the other model parameters, but allowed uncertainty in the chronically infected population size to be correctly estimated.

### Posterior distribution sampling

Sampling from the posterior distributions for all parameters was carried out via MCMC simulation using OpenBUGS version 3.2 [30] and the BRugs package for R [31], within the R statistical computing environment (version 3.0.3) [32]. Two independent chains were run for 10,000 iterations, with the first 5,000 samples discarded as burn-in. Graphical methods were used to establish convergence of the chains.

### Sensitivity analyses

Additional simulations were conduct to investigate the sensitivity of *P*(HCV+) and *P*(PWID|HCV+) to assumptions regarding parameter values used in the multi-state Markov model of PWID population size. Two plausible alternative values for the average duration of injection career, and two alternatives for excess mortality were tested (see Additional file 1: Appendix C).

## Results

**Figure 2:**
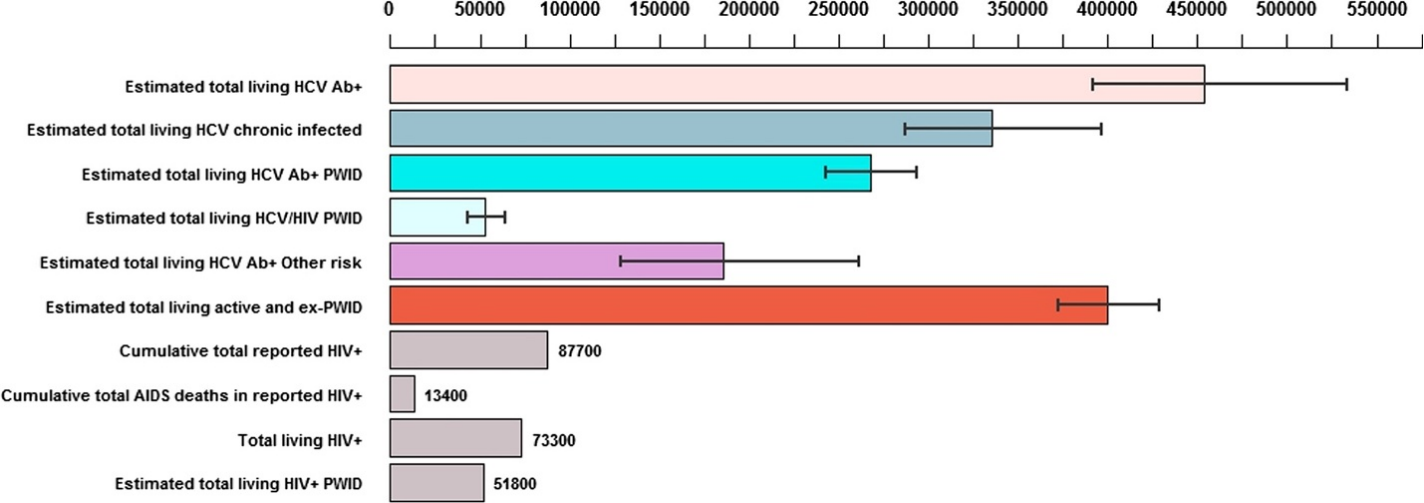
**Epidemiological situation for HIV and HCV infection in Malaysia, as of end 2009.** Capped lines indicate 95% credible intervals.

**Figure 3:**
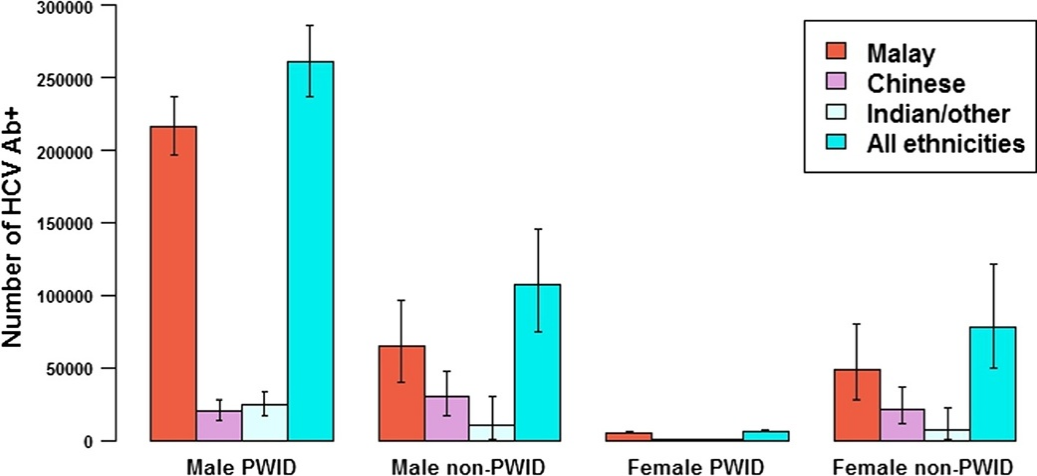
**Estimated prevalent number of HCV Ab+ infected persons according to ethnicity, sex, and mode of transmission.** Capped lines indicate 95% credible intervals.

An estimated 453,700 people (95% credible interval [CrI]: 391,700-535,100) were living with HCV infection in Malaysia in 2009 (2.5% of the population aged 15-64 years), of whom 59% (95% CrI: 50-68%) acquired their infection through injecting. The total population size with chronic HCV infection is estimated at 335,200 (95% CrI: 287,500-396,800), which is more than three times the number of people reported to be living with HIV in 2009 (Figure 2). These aggregate figures conceal variation according to sex and ethnicity; the estimated prevalence in females (1.0%, 95% CrI: 0.6-1.4%) is considerably lower than among males (4.0%, 95% CrI: 3.6-4.5%), and the estimated number of HCV Ab+ male Malays (281,400, 95% CrI: 249,400-318,300) is much higher than the numbers for the other two ethnicity groups (50,200 and 35,000 male Chinese and male Indian/other, respectively) (Figure 3). The estimated adult population prevalences according to ethnicity group are 2.9%, 1.1%, and 0.6%, for Malays, Chinese, and Indian/other, respectively. The prevalences according to ethnic group do not closely follow the ethnicity proportions in the general population, because of differences in the prevalence of PWID and the prevalence of HCV infection in PWID between ethnic groups.

The posterior estimate for the female:male prevalence ratio among those with non-PWID risk was 0.73 (95% CrI: 0.53-0.98), yielding sex-specific estimates of the infected population with non-PWID risk of 106,900 and 78,050, for males and females, respectively. The estimated percentage of all HCV Ab+ persons whose infection is attributable to non-PWID transmission is 41% (95% CrI: 32-50%).

### Sensitivity to *P*(PWID)

The results of simulations using alternative values for the average duration of injecting (which defined the cessation probability) and for excess mortality when modelling the PWID population size indicated that the main outcome - the overall prevalence of HCV - was not overly sensitive to the original choices for these parameters. The range of estimates for the median population prevalence of HCV produced by the sensitivity analyses was 2.15 to 2.83% (see Additional file 1: Appendix C).

## Discussion

This study is the first to quantify the epidemiological situation in Malaysia at the national level in terms of prevalence and distribution over modes of transmission. By combining all of the available - albeit sparse - evidence within a single statistical framework, we were able to estimate the epidemiological parameters of interest, and uncertainty around these parameters. Approximately 454,000 persons (2.5% of 15-64 year-olds [33]) were estimated to be living with HCV infection in 2009. Although the majority of HCV infections occur in PWID, the non-PWID risk group still represents a large proportion (41%) of the HCV Ab+ population; this situation differs from that in countries such as Australia and the UK, where 80% or higher of prevalent infections are attributable to injecting drug use [15],[34],[35].

Stratification by sex and ethnicity was revealing; given that differences in HCV Ab+ prevalence between Malay PWID - in whom the highest prevalence was estimated - and the other ethnic groups may be associated with ethnic differences in injecting behaviours, and given that only a small percentage of women are PWID, the estimated HCV Ab+ prevalence among strata does not simply mirror the sex and ethnicity distribution in the population.

Our estimated overall prevalence of 2.5% in persons aged 15-64 years is consistent with the findings of the Global Burden of Disease (GBD) 2010 study, which estimated HCV Ab+ prevalence at the GBD region level for the year 2005 using a hierarchical modelling approach [36]. For the Southeast Asia GBD region, overall prevalence was estimated at 2.0% (95% CI of 1.7-2.3). Within the countries of the WHO Western Pacific Region (WPR), Malaysia occupies an upper tier in terms of HCV prevalence; of the WPR countries with prevalence estimates available, it is comparable to Vietnam, with a population prevalence of 2-2.9% [37] but lower than China (3.2% [9]).

Despite the strengths of the modelling approaches used, this study has a number of limitations. We emphasise that deriving estimates of epidemiological parameters from multiple data sources depends heavily on the degree of accuracy, representativeness, and bias in those sources; in the case of sparse available data (such as the situation in Malaysia), these factors are even more crucial for producing valid estimates. Additional data informing any of the key parameters may result in revised estimates.

The second limitation concerns the extremely simple model of the prevalent number of active and ex-PWID. The cessation probability (moving from active to ex-PWID) and the relapse probability were not based on evidence, and the probability of becoming an active PWID was fitted to a single data point. The dynamic model of the PWID population also disregards individual heterogeneity in the drug-using population regarding age at commencement, age at cessation, and mortality risk. The obtained ratio of ex- to active PWID (1.34 to 1) is comparable to the ratio estimated for England (1.95, 95% CrI: 0.72-4.81) using a different approach and informed by much more data [38]; however, the similarity to the ratio for England cannot be considered as validation. Despite the assumptions made for the parameters of the PWID population model, sensitivity analyses indicated - for cessation probability and excess mortality at least (see Additional file 1: Appendix C) - that the principal result was not unduly influenced by the parameter values chosen.

Our estimate of prevalence within the non-PWID population depends on the testing of blood donors carried out more than 20 years previously [19]. There are two points of concern regarding the use of this data source to inform the prevalence in non-PWID. First, despite the study authors taking care to ensure that donors were volunteers and that individuals with high-risk behaviours were excluded, obtained seroprevalence values nevertheless could reflect inclusion of some individuals with a history of injecting drugs. Second, although we adjusted for the specificity of the second generation assays used at that time, we have no way to determine if the prevalence in this population has increased, decreased, or remained stable since. A study among blood donors conducted in 2008-2009 in north-eastern Malaysia reported a HCV seroprevalence of 0.45% [20], but donor pre-screening for blood-borne virus risk factors has become much more stringent compared with the early 1990s. However, in the absence of new data, one could investigate the impact on results when other plausible values are substituted for this parameter. Further sensitivity analyses could be useful for examining the impact on parameter estimates from alternative assumptions about the data.

Finally, the uncertainty associated with the model parameters - although correctly propagated from all data sources/parameters concerned - is somewhat under-estimated due to the lack of a realistic level of uncertainty around the prevalence of active and ex-injectors (*P*(PWID)).

## Conclusion

The prevalent number of individuals living with HCV infection in Malaysia is estimated to be very high. To reduce the considerable - and inevitable - future public health and economic burden due to morbidity and mortality from severe liver-related disease, current prevention and screening initiatives and the numbers of chronically infected individuals receiving antiviral treatment will need to be drastically scaled up. The proposed methodology may be of value for other countries with a limited evidence base, and for whom HCV infection is a growing public health issue.

## Additional file

## Electronic supplementary material

Additional file 1: Appendices A, B, and C. Detailed description of age-structured multi-state Markov model for estimating PWID population size, detailed description of equations specifying the evidence synthesis model, and methods for and results of sensitivity analyses. (DOCX 241 KB)

Below are the links to the authors’ original submitted files for images.

Authors’ original file for figure 1

Authors’ original file for figure 2

Authors’ original file for figure 3
