# Supplementary material for: Bridging the data gaps in the epidemiology of hepatitis C virus infection in Malaysia using multi-parameter evidence synthesis
Source: BMC Infect Dis. 2014 Nov 7;14:564. doi: 10.1186/s12879-014-0564-6 (PMC4229598; doi:10.1186/s12879-014-0564-6)
Supplement: Supplementary file 1 — Additional file 1: Appendices A, B, and C. Detailed description of age-structured multi-state Markov model for estimating PWID population size, detailed description of equations specifying the evidence synthesis model, and methods for and results of sensitivity analyses. (DOCX 241 KB) [file 12879_2014_564_MOESM1_ESM.docx]

**Additional File**

*Title:*

Bridging the data gaps in the epidemiology of hepatitis C virus infection in Malaysia using multi-parameter evidence synthesis

*Authors:*

Scott A. McDonald, Rosmawati Mohamed, Maznah Dahlui, Herlianna Naning, Adeeba Kamarulzaman

*BMC Infectious Diseases, 2014*

**APPENDIX A – ESTIMATION OF THE PWID POPULATION SIZE**

The numbers of active and ex-PWID in 2009 was estimated using a simple individual-based multi-state Markov model representing the non-PWID, active PWID, and ex-PWID subpopulations. Fig. A1 shows the model structure, in which three compartments represent the entire Malaysian population aged 15 years and older.

The following is a brief description of model function. Every simulation year, β 15-year olds enter the non-PWID compartment. For those non-PWID aged 15 through 39 years only, there is a fixed annual transition probability (*P*_α_) of becoming an active PWID (i.e., transitioning from the non-PWID to the active PWID compartments), and a fixed annual probability of ceasing active injecting (*P*_γ_) and so move to the ex-PWID compartment. Ex-PWID can ‘relapse’ (move back to the active PWID compartment) with a fixed annual probability (*P*_κ_). Ageing is taken into consideration, so that the total size of both active and ex-PWID sub-populations for a given age-range at a given time can be estimated. One-year width age groups ranged from 15 years to 64 years. After their 49^th^ year, all active PWID are assumed to cease injecting, and no relapses can occur. Mortality is simulated by applying the national age-specific all-cause mortality rates for the year 2000 [1] to all compartments (*P*_μ_), and excess mortality (due to overdose, trauma, injury, suicide, etc) of 13 times the general population rate is assumed for active PWID only; this is the standardised mortality ratio estimated for problem illicit drug users aged 15-54 years [2].

The cessation probability was set assuming an average active injecting duration of 15 years (= 1 – *e*^-1/15^), and the relapse probability was converted from the monthly relapse rate from a modelling study of PWID in Amsterdam (0.004/month) [3] to an annual probability (= 1 – *e*^-0.004*12^). The annual transition probability of becoming an active injector was then adjusted so that the simulated active PWID population size in 2009 fitted a single existing data point, namely the prevalent number of active PWID in 2009 (170,000), which was derived by experts’ consensus [4].

The starting year of the simulation was set arbitrarily to the year 1950, and time-invariant initiation, cessation, and relapse parameter values were applied over the simulation period 1950-2009. For simplicity, the age-specific mortality rates for 2000 [1] were applied to all years, and population growth and other demographic changes were not considered. Also for simplicity, the initial age-specific population sizes for 1950 were set to the 2009 values [5]. Because of the constant total population size, the modelled prevalent number of active PWID and ex-PWID settle to stable values.

Uncertainty in the model-generated annual numbers of new active PWID was simulated as in previous research [6], by assuming that the number of new active PWID is produced by a Poisson process, in which case variability can be expressed by a Gamma distribution with shape parameter set to the simulated annual new active PWID and with scale parameter set to 1 (where *New*_y_ is the number of new active PWID in year *y*, and *N*_y-1_ is the number of non-PWID at the end of year *y-*1):

*New_y_  = (N*_y-1_ *∙ P*_α_*)*

*E[New_y_] ~ Gamma*(*New_y_*, 1)

The uncertainty range was then defined as the 5th and 95th percentiles of this distribution. The multi-state Markov model predicted a prevalent total number of 399,200 PWID aged 15-64 years in 2009 (of whom 228,300 were ex-PWID). Given the 2009 national mid-year population size for this age group, 18,005,800, [5] the corresponding PWID prevalence was therefore estimated at 2.22%, with uncertainty range of 2.02 to 2.30% (derived as described in the preceding paragraph). The obtained ratio of ex-PWID to active PWID was therefore 1.35 to 1.

**Table A1.** Parameters of the multi-state Markov model of the PWID population in Malaysia.

| **Parameter** | **Value** | **Source/assumption** |
| --- | --- | --- |
| β – annual number of 15-year olds entering the population  *P*_α_ – annual transition probability, non-PWID to active PWID  *P*_γ_ – annual transition probability, active PWID to ex-PWID  *P*_κ_ – annual transition probability of relapse, ex-PWID to active PWID  Ω – excess mortality  *P*_μ_ – annual mortality probability | 524,520  1 – *e*^-0.00115^  (15-39 years only)  1 – *e*^-1/15^  (15-49 years)  1  (50+ years)  1 – *e*^-0.004*12^  13  (active PWID)  1  (ex-PWID)  1 – *e*^-µΩ^, where  µ=0.00093  (15-19 years)  µ=0.00118  (20-24 years)  µ=0.00132  (25-29 years)  µ=0.00160  (30-34 years)  µ=0.00215  (35-39 years)  µ=0.00310  (40-44 years)  µ=0.00433  (45-49 years)  µ=0.00767  (50-54 years)  µ=0.01194  (55-59 years)  µ=0.02131  (60-64 years) | Estimated from published national demographic data for 15-19 years [5]  Derived by fitting to estimated prevalent active PWID in 2009 (170,000)[4]  Assumed average active injecting career of 15 years  Relapse rate of 0.004/month, from modelling study [3]  Standardised mortality ratio, from study of problem illicit drug users [2]  Converted from WHO national mortality rates for the year 2000 [1] |

**Figure A1.** Multi-state Markov model of the Malaysian population aged 15-64 years.


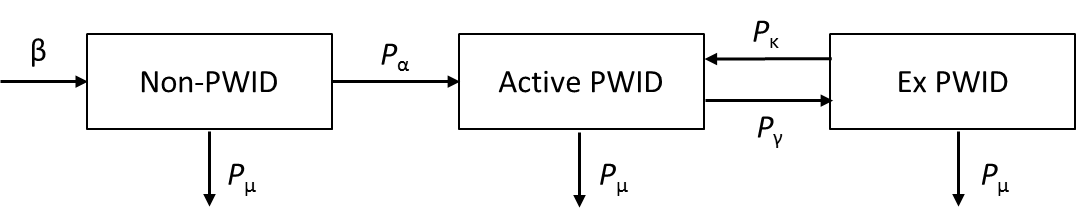


**APPENDIX B – MPES MODEL EQUATIONS**

All model inputs and estimated values are with respect to the adult population aged 15-64 years only, and are assumed to apply to the year 2009. Below, we use the symbol ρ for proportion parameters, and π for HCV Ab+ prevalence parameters. In all instances in which a prior distribution was specified, the appropriate prior for the likelihood was selected (e.g., beta prior for binomial likelihood).

***P*(PWID|HCV+): probability of PWID risk among HCV Ab+ persons**

The probability of PWID risk given HCV Ab+ status, *P*(PWID|HCV+), can be computed through multiplication of the component probabilities of Bayes’ Theorem (see Eq. 1 in main paper). We equivalently estimated the proportion of HCV Ab+ individuals with PWID risk as marginal quantities: the estimated number of PWID stratified by sex and ethnicity divided by the estimated [stratified] number of HCV Ab+ persons [7]:

$$\rho_{PWID,sex,ethnicity}=\frac{N_{PWID,sex,ethnicity}}{N_{PWID,sex,ethnicity}+ N_{nonPWID,sex,ethnicity}}$$

The proportion with non-PWID risk is calculated analogously: the estimated [stratified] number of HCV Ab+ persons with non-PWID risk divided by the estimated [stratified] number of HCV Ab+ persons:

$$\rho_{nonPWID,sex,ethnicity}=\frac{N_{nonPWID,sex,ethnicity}}{N_{PWID,sex,ethnicity}+ N_{nonPWID,sex,ethnicity}}$$

A weighted average for both proportions can also be computed by summing over strata in the terms in the numerator and denominator.

***P*(HCV+|nonPWID): probability of HCV in persons with non-PWID risk**

A binomial distribution was specified for the probability of HCV infection in non-PWID, and assigned an vague beta prior. The seroprevalence parameter, π_Obs_, was informed by ethnicity-specific data from male blood donors only (see Table 1 in main article), where *x* is the number of positive cases out of *n* specimens tested:

$$\pi_{Obs} \sim Beta\left( 1,1 \right)$$

$$x \sim Binomial\left( n,\pi_{Obs} \right)$$

The observed seroprevalence was then adjusted for the test specificity (*Spec =* 99.7%) to estimate the true seroprevalence in male non-PWID, π_Male_, according to the functional relationship between observed and true prevalence (see also Reference [8)):

$$\pi_{Obs}=\pi_{nonPWID,Male}+\left( 1-\pi_{nonPWID,Male} \right)(1-Spec)$$

To estimate the seroprevalence in female non-PWID, a functional relationship was defined: seroprevalence in males was multiplied by the female:male positivity ratio ($\psi$) informed by the relevant counts from two blood donor studies in neighbouring countries (see Table 1):

$$\psi\sim Beta\left( 1,1 \right)$$

$$y_{1} \sim Binomial\left( z_{1},\psi\right)$$

$$y_{2} \sim Binomial\left( z_{2},\psi\right)$$

$$\pi_{nonPWID,Female}=\pi_{nonPWID,Male} \psi$$

***P*(HCV+|PWID): probability of HCV in PWID**

A single study informed the probability of HCV infection in male PWID, stratified by ethnicity; these parameters were specified as binomial distributions with vague priors, where *n* is the number of PWID and *x* is the number infected:

$$\pi_{PWID} \sim Beta\left( 1,1 \right)$$

$$x \sim Binomial\left( n,\pi_{HCV|PWID} \right)$$

The probability of female PWID being HCV Ab+ was assumed to be identical.

***P*(PWID): probability of PWID in the general population**

The population proportion of PWID was specified as an informative beta prior distribution, Beta(1275, 28692); this corresponded to the multi-state Markov model-derived PWID prevalence for males, assuming 97.6% of all PWID are males. For females, the prior distribution was set to Beta(940, 869558), which encodes the assumption that 2.4% of all PWID are females.

Stratification by ethnicity was achieved by specifying functional nodes: the estimated proportion of PWID represented by each ethnic group, φ_ethnicity_, was assumed to be binomially distributed and – in the absence of any data – identical for males and females. This proportion was then multiplied by the sex-specific prevalence of PWID and divided by the national ethnicity proportion [5] to give the stratified population PWID prevalence; e.g., for males:

$$\rho_{PWID,male} \sim Beta\left( 1275,28692 \right)$$

$$x_{PWID,ethnicity} \sim Binomial(n_{PWID,ethnicity},\phi_{ethnicity})$$

$$\rho_{PWID,male,ethnicity}=\rho_{PWID,male} ⨉ \phi_{ethnicity}/{natprop}_{ethnicity}$$

***P*(HIV+|HCV+): prevalence of HIV coinfection in HCV Ab+ persons**

The counts underlying this prevalence value were incorporated as an additional binomially distributed parameter in the MPES model, where *n* is the number of HCV-infected persons and *x* is the number coinfected with HIV:

$$\pi_{HIVcoinf} \sim Beta\left( 1,1 \right)$$

$$x \sim Binomial\left( n,\pi_{HIVcoinf} \right)$$

**Prevalence of chronic HCV infection**

Finally, the number of persons with chronic HCV infection was computed by specifying an informative beta prior derived from the point estimate (74%) and 95% confidence interval reported in a recent systematic review [9], and multiplying by the total estimated HCV Ab+ population size (*N_Ab+_*):

$$\nu_{chronic} \sim Beta\left( 446,157 \right)$$

$$N_{chronic}=\nu_{chronic} N_{Ab+}$$

**Table B1.** MPES model parameters with specified prior distribution and rationale for or evidence informing the parameter.

| **Parameter** | **Distribution/ functional form** | **Rationale/evidence** |
| --- | --- | --- |
| π*_PWID,Malay_* π*_PWID,Chinese_* π*_PWID,other_*  π*_nonPWID,male,Malay_* π*_nonPWID,male,Chinese_* π*_nonPWID,male.other_*  Ψ (female to male ratio)  *Spec*  ρ*_PWID,male_*  ρ*_PWID,female_*  ν*_chronic_*  π*_HIVcoinf_* | Beta(1,1)  Beta(1,1)  Beta(1,1)  Beta(1,1)  Beta(1,1)  Beta(1,1)  Beta(1,1)  99.7%  Beta(1275, 28692)  Beta(940, 869558)  Beta(446,157)  Beta(1,1) | Vague priors; informed by direct evidence from study of HCV-positive male PWID, stratified by ethnicity.  Vague priors; informed by direct evidence from study of HCV-positive male blood donors (stratified by ethnicity), and reported test specificity.  Vague prior, informed by sex ratio of HCV-positive blood donors in two neighbouring countries (Thailand and Singapore)  Reported specificity of 2^nd^ generation HCV assay.  Informative priors constructed from model-estimated active and ex-PWID prevalence, assuming 97.6% males and 2.4% females.  Prior informed by systematic review of longitudinal studies of viral clearance.  Vague prior; informed by direct evidence from study of HIV/HCV coinfection in Malaysian fisherman community. |

**APPENDIX C – SENSITIVITY ANALYSES**

One advantage of the MPES methodology is that if uncertainty in parameters informed by data sources can be quantified appropriately, the uncertainty in the parameters of interest is correctly propagated from all other model parameters. In this sense, conventional sensitivity analysis is not needed to address uncertainty, because uncertainty is already in the model. However, if there is reason to believe that a particular data source is biased, but there is no information on the extent or the direction of the bias, or if two potential data sources give conflicting parameter estimates, then a sensitivity analysis could be useful. In addition, if a parameter value is adopted in the absence of any supporting data, the sensitivity to this choice can be explored by comparison with the results obtained if other, also reasonable, values for the same parameter are selected.

As the value of the parameter *P*(PWID) supplied to the MPES model depends on the predictions of the multi-state Markov model for the PWID population size (specifically, the predicted size of the ex-PWID population) in 2009, *P*(PWID) is therefore sensitive to:

- The average injecting career length (for which 15 years had been assumed). No data were available for the Malaysian PWID setting; other values may be equally plausible. We therefore tested two additional average career lengths (10 years and 20 years) in sensitivity analysis (i).
- The mortality rates in both active PWID and ex-PWID. The default excess mortality of 13 times background mortality [2] assumed for active PWID, although obtained from a study of illicit drug users, may be unrealistically high for the Malaysian setting. It also may not be plausible to assume a large degree of excess mortality in active-PWID but none in ex-PWID, because both groups may have detrimental health-influencing behaviours in common other than drug injecting. We therefore tested the impact of (a) reducing the excess mortality in active-PWID to half the default value (i.e., 6.5 times background mortality), and (b) setting ex-PWID mortality to the same value as for active-PWID (13 times background mortality [2]) in sensitivity analysis (ii).

**Methods**

***Sensitivity analysis (i)***

The procedure of fitting the free transition probability parameter (*P*_α_) to the estimated number of active PWID in 2009 (as described above in Appendix A) was repeated twice further, using two different values for the average injecting career length (10 years and 20 years), to obtain new estimates of *P*(PWID), with uncertainty interval derived using the Gamma method (described above in Appendix A). Then, the MPES model was run using each of the two new *P*(PWID) estimates, to produce posterior estimates of the parameters of interest – *P*(HCV+) and *P*(PWID|HCV+) – stratified by sex and ethnicity.

***Sensitivity analysis (ii)***

The procedure of fitting the parameter (*P*_α_) to the estimated number of active PWID in 2009 was carried out under two alternative assumptions regarding excess mortality. The first run tested the impact of assuming 50% of the excess mortality in active PWID compared with the default value of 13 [2] (i.e., 6.5 times background mortality rates), with no excess mortality in ex-PWID. The second run assumed that ex-PWID had the same excess mortality as active PWID (i.e., both had 13 times background mortality rates). Separate runs of the MPES model using the resulting two new *P*(PWID) estimates produced posterior estimates of the parameters of interest.

**Results**

***Sensitivity analysis (i)***

Assuming 10 years as the average duration of injecting career, the prevalent ex-PWID population size was predicted to be 313,900 in 2009, yielding an overall (ex- and active) PWID prevalence of 2.69% of the population aged 15-64 years. The obtained ratio of ex-PWID to active PWID was 1.84 to 1. Fig. C1 shows the corresponding impact on *P*(HCV+).

Assuming 20 years as the average duration of injecting career, the prevalent ex-PWID population size was predicted to be quite a bit smaller (185,200) in 2009, reflecting the longer average time spent as an active injector. This yielded ratio of ex- to active PWID was 1.08 to 1 (Table C1); the overall PWID prevalence was estimated at 1.98% of the population aged 15-64 years.

***Sensitivity analysis (ii)***

For the case where excess mortality among active PWID was reduced by 50%, the prevalent ex-PWID population size was predicted to be 238,400 in 2009, yielding an overall PWID prevalence of 2.27% of the 15-64 years population. The obtained ratio of ex-PWID to active PWID was 1.39 to 1 (Table C1). Fig. C1 displays the corresponding effects on *P*(HCV+).

If both active PWID and ex-PWID are assumed to have the same excess mortality (13 times), the ex-PWID population is much reduced (136,500), with a corresponding lower ex- to active PWID ratio (0.80), and overall PWID prevalence among 15-64 year-olds estimated at 1.65%, but impact on the overall HCV prevalence is small (Table C1).

**Table C1.** Description of multi-state Markov model parameter values that were varied in the two sensitivity analyses, with resulting ex-PWID population size and the ratio ex-PWID to active PWID estimated for 2009. The fourth and fifth columns show the evidence synthesis model results for two parameters of interest, as median posterior probabilities (with 95% credible interval).

| **Variant** | **Total  ex-PWID** | **Ratio ex- to active** | **P(HCV**+**) [95% CrI]** | **P(PWID\|HCV**+**) [95% CrI]** |
| --- | --- | --- | --- | --- |
| *Sensitivity analysis (i)*  - Average length of injecting career  shorter (=10 years)  - Average length of injecting career  longer (=20 years)  *Sensitivity analysis (ii)*  - Excess mortality in active PWID  smaller (=6.5 years)  - Excess mortality in active PWID  and ex-PWID identical (=13 years) | 313,900  185,200  238,400  136,500 | 1.84  1.08  1.39  0.80 | 2.83%  [2.46-3.28%]  2.35% [2.00-2.79%]  2.55%  [2.19-3.00%]  2.15%  [1.79-2.59%] | 0.60  [0.51-0.69]  0.52  [0.43-0.61]  0.64  [0.55-0.72]  0.56  [0.47-0.66] |


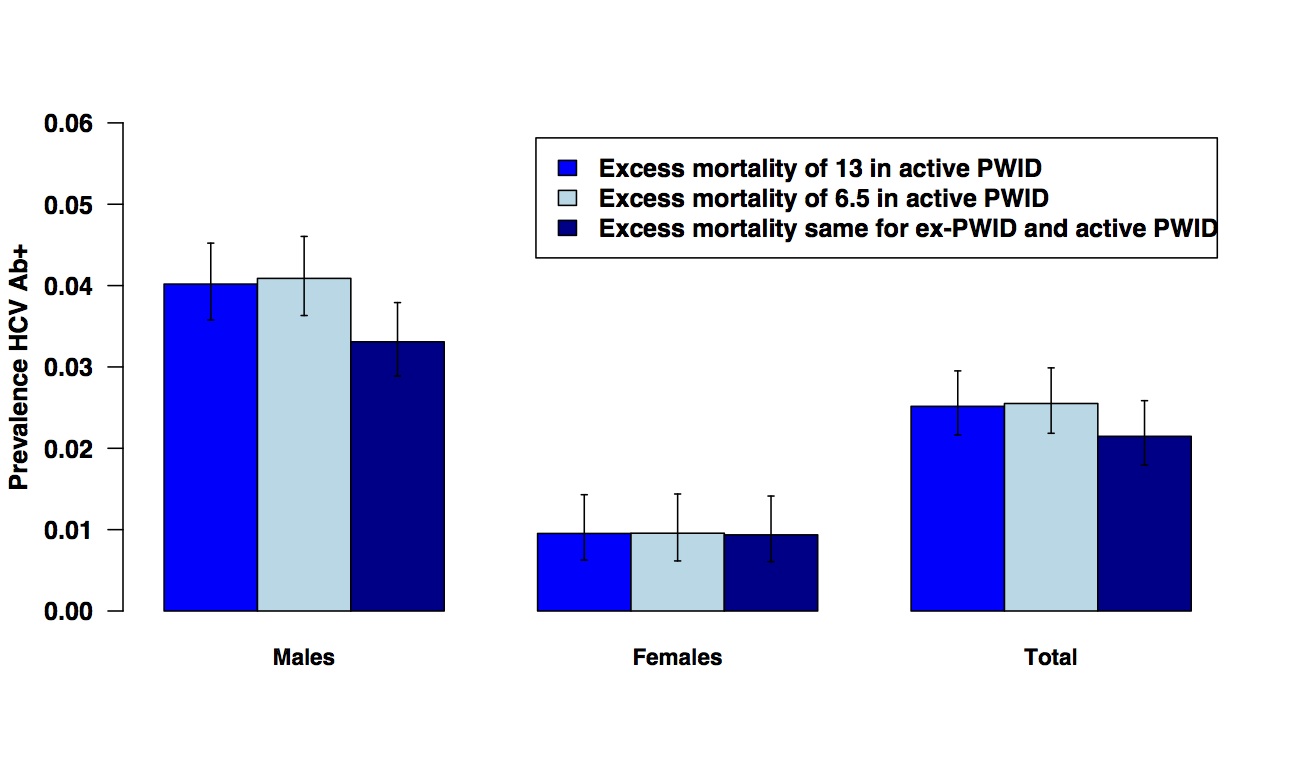

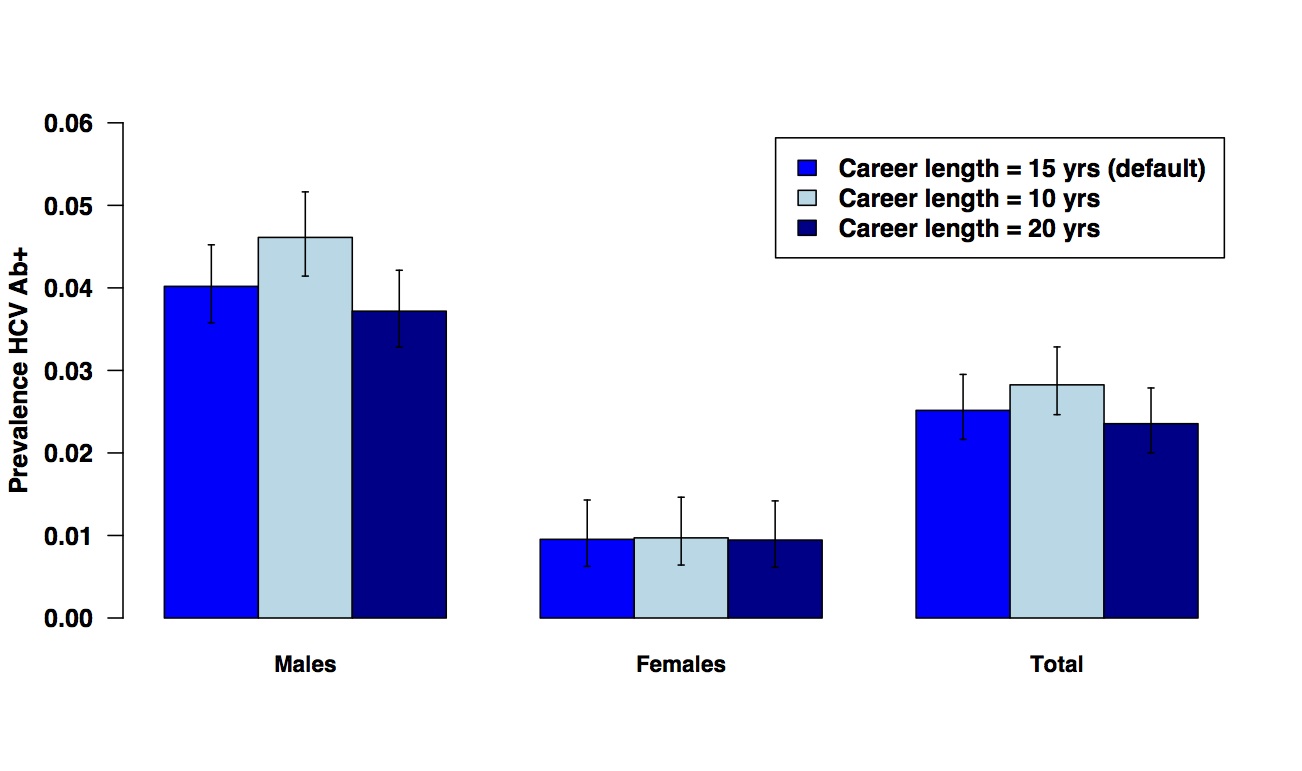
**Figure C1.** Results of sensitivity analysis (i), in which average injecting career length was varied (top panel), and sensitivity analysis (ii), in which the degree of excess mortality in active PWID and/or ex-PWID was varied (bottom panel). Point estimates and 95% credible intervals are shown for the main parameter of interest, stratified by sex: the probability of being HCV antibody-positive.

**REFERENCES**

**[1] Global Health Observatory Data Repository [http://apps.who.int/gho/data/?theme=main&vid=60990].**

[2] Degenhardt L, Hall W, Warner-Smith M, Lynskey M: **Illicit drug use**. *Comparative Quantification of health risks: Global and regional burden of disease attributable to selected major risk factors*. Edited by Ezzati M. et al. Geneva: World Health Organization; 2004:1109-1176.

[3] de Vos AS, van der Helm JJ, Matser A, Prins M, Kretzschmar ME: **Decline in incidence of HIV and hepatitis C virus infection among injecting drug users in Amsterdam; evidence for harm reduction?** *Addiction* 2013, **108:**1070-1081.

[4] Ministry of Health Malaysia and WHO: *Estimation and projection of the HIV epidemic Malaysia 2009*. National Consensus Workshop, 26–29 May 2009.

[5] Department of Statistics Malaysia: *Yearbook of Statistics Malaysia*. Kuala Lumpur: Department of Statistics; 2009.

[6] Haagsma JA, van der Zanden BP, Tariq L, van Pelt W, van Duynhoven YTPH, Havelaar AH: **Disease burden and costs of selected foodborne pathogens in the Netherlands, 2006**. Bilthoven, the Netherlands: National Institute for Public Health and the Environment; 2009

[7] De Angelis D, Sweeting M, Ades A, Hickman M, Hope V, Ramsay M: **An evidence synthesis approach to estimating Hepatitis C prevalence in England and Wales**. *Stat Methods Med Res* 2009, **18:**361-379.

[8] Lew RA, Levy PS: **Estimation of prevalence on the basis of screening tests.** *Stat Med* 1989, **8:**1225-1230.

[9] Micallef JM, Kaldor JM, Dore GJ: **Spontaneous viral clearance following acute hepatitis C infection: a systematic review of longitudinal studies**. *J Viral Hepat* 2006, **13:**34-41.
