# Supplementary figures and images for: Bridging the data gaps in the epidemiology of hepatitis C virus infection in Malaysia using multi-parameter evidence synthesis
Source: BMC Infect Dis. 2014 Nov 7;14:564. doi: 10.1186/s12879-014-0564-6 (PMC4229598; doi:10.1186/s12879-014-0564-6)

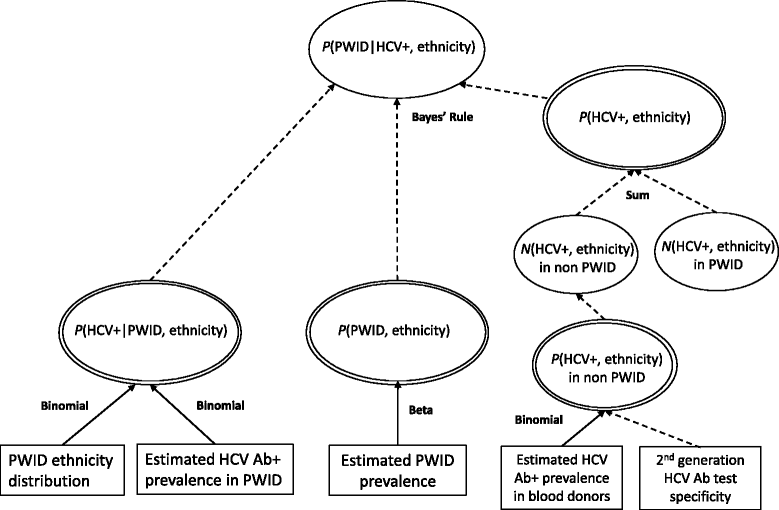

Supplement: Supplementary file 2 — Authors’ original file for figure 1 [file 12879_2014_564_MOESM2_ESM.gif]

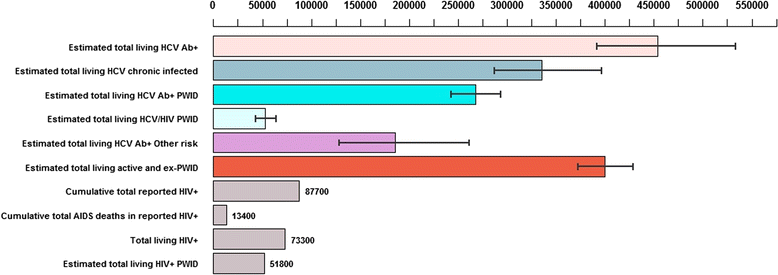

Supplement: Supplementary file 3 — Authors’ original file for figure 2 [file 12879_2014_564_MOESM3_ESM.gif]

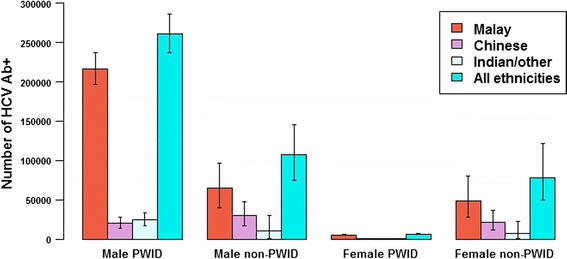

Supplement: Supplementary file 4 — Authors’ original file for figure 3 [file 12879_2014_564_MOESM4_ESM.gif]
